# Unenhanced abdominal low-dose CT reconstructed with deep learning-based image reconstruction: image quality and anatomical structure depiction

**DOI:** 10.1007/s11604-022-01259-0

**Published:** 2022-03-14

**Authors:** Tetsuro Kaga, Yoshifumi Noda, Takayuki Mori, Nobuyuki Kawai, Toshiharu Miyoshi, Fuminori Hyodo, Hiroki Kato, Masayuki Matsuo

**Affiliations:** 1 Department of Radiology, Gifu University, 1-1 Yanagido, Gifu, 501-1194 Japan; 2 Department of Radiology Services, Gifu University Hospital, 1-1 Yanagido, Gifu, 501-1194 Japan; 3 Department of Radiology, Frontier Science for Imaging, Gifu University, 1-1 Yanagido, Gifu, 501-1194 Japan

**Keywords:** Unenhanced abdominal low-dose CT, Deep learning-based image reconstruction, Abdominal anatomical structures depiction, CT dose-index volume, Size-specific dose estimates

## Abstract

**Purpose:**

To evaluate the utility of deep learning-based image reconstruction (DLIR) algorithm in unenhanced abdominal low-dose CT (LDCT).

**Materials and methods:**

Two patient groups were included in this prospective study: 58 consecutive patients who underwent unenhanced abdominal standard-dose CT reconstructed with hybrid iterative reconstruction (SDCT group) and 48 consecutive patients who underwent unenhanced abdominal LDCT reconstructed with high strength level of DLIR (LDCT group). The background noise and signal-to-noise ratio (SNR) of the liver, pancreas, spleen, kidney, abdominal aorta, inferior vena cava, and portal vein were calculated. Two radiologists qualitatively assessed the overall image noise, overall image quality, and abdominal anatomical structures depiction. Quantitative and qualitative parameters and size-specific dose estimates (SSDE) were compared between SDCT and LDCT groups.

**Results:**

The background noise was lower in LDCT group than in SDCT group (*P* = 0.02). SNRs were higher in LDCT group than in SDCT group (*P* < 0.001–0.004) except for the liver. Overall image noise was superior in LDCT group than in SDCT group (*P* < 0.001). Overall image quality was not different between SDCT and LDCT groups (*P* = 0.25–0.26). Depiction of almost all abdominal anatomical structures was equal to or better in LDCT group than in SDCT group (*P* < 0.001–0.88). The SSDE was lower in LDCT group (4.0 mGy) than in SDCT group (20.6 mGy) (*P* < 0.001).

**Conclusions:**

DLIR facilitates substantial radiation dose reduction of > 75% and significantly reduces background noise. DLIR can maintain image quality and anatomical structure depiction in unenhanced abdominal LDCT.

## Introduction

Unenhanced computed tomography (CT) has become indispensable in modern medicine in applications such as screening and diagnosis of acute diseases, assessment of therapeutic response, tumor recurrence, and follow-up for various patients’ conditions [1]. However, repeated CT examinations can lead to excessive medical radiation exposure, which increases the risk of adverse events to clinical staff and patients [2]. Radiation dose reduction strategies have always been a high priority issue in CT examinations. However, they tend to facilitate increased image noise, which can deteriorate image quality and diagnostic performance [3].

Image reconstruction techniques for the acquisition of CT images have been remarkably evolved in recent years [4]. For example, the statistical iterative reconstruction (IR) technique, especially the hybrid-IR technique, has managed to reduce image noise without image quality degradation compared with filtered back projection (FBP) [5]. Consequently, hybrid-IR has now become widely used in clinical setting. Hybrid-IR technique had been applied to low-dose CT (LDCT) protocol because it could significantly reduce image noise. However, LDCT images reconstructed with hybrid-IR were inferior to standard-dose CT (SDCT) images reconstructed with FBP in terms of detectability of low-contrast lesions and spatial resolution [6–9]. The detectability of low-contrast lesion was crucial for diagnostic imaging, as a result, the clinical use of abdominal LDCT has been limited.

Recently, the deep learning-based image reconstruction (DLIR) technique has been introduced as a next-generation CT image reconstruction method [10, 11]. Noda et al. [12, 13] have reported the impact of DLIR on whole-body and abdominal contrast-enhanced LDCT protocols, and achieved extremely low radiation dose (2.9 mGy and 2.3 mGy in CT dose-index volumes [CTDI_vol_]), while maintaining effective image quality and lesion detectability. Jensen et al. [14] reported that contrast-enhanced LDCT reconstructed with DLIR achieve 65% radiation dose reduction and while maintaining the detectability of liver lesions compared to SDCT reconstructed with FBP. To the best of our knowledge, however, no study has evaluated the use of a DLIR in an unenhanced abdominal LDCT protocol. Therefore, in this study, we aim to evaluate the usefulness of the DLIR technique in unenhanced abdominal LDCT protocol for the assessment of image quality and to compare with SDCT reconstructed using hybrid-IR technique.

## Materials and methods

### Phantom study

We performed CT scans of the self-made phantom to assess the objective image quality. This phantom study was conducted under the same scan protocols as shown in “Imaging technique” section without dose modulation, and noise power spectrum (NPS) and modulation transfer function (MTF) were calculated using CT measure software (version 0.98f; Japanese Society of CT Technology, Hiroshima, Japan). NPS was calculated by the radial frequency method with a square circular regions of interest (ROI) [15] on CT image of self-made water phantom. MTF was calculated by circular edge technique [16] using a self-made phantom in which acrylic resin rod was immersed in water.

### Human clinical study

**Fig. 1:**
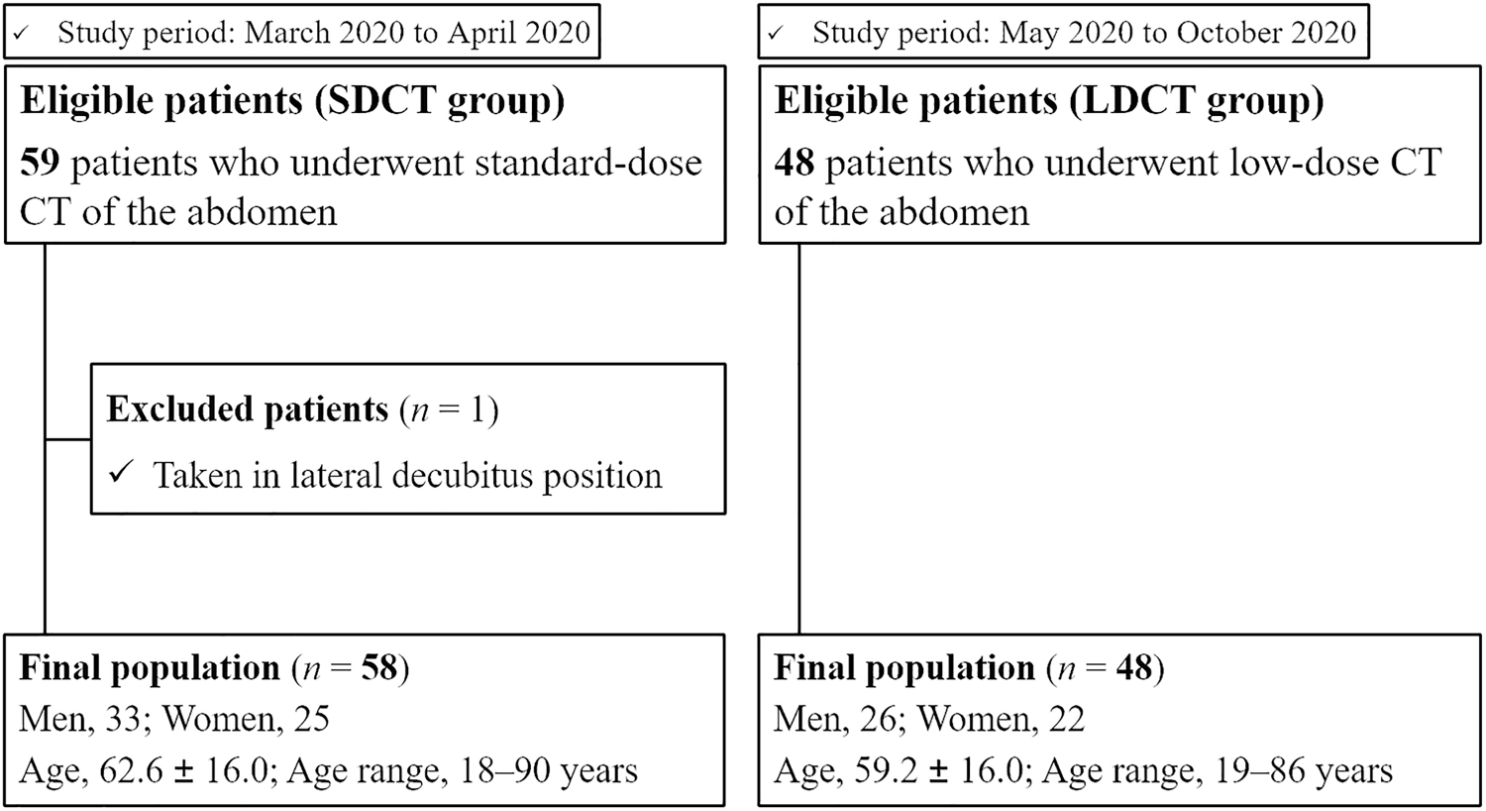
Flow chart of the included and excluded patients

This prospective study was approved by our Institutional Review Board. This study consisted of two patient group sets (Fig. 1). The first group initially included 59 consecutive patients who underwent unenhanced abdominal SDCT from March 2020 to April 2020; however, one patient was excluded because the CT scan was performed in the lateral decubitus position. Thus, the remaining 58 patients were included in this study (SDCT group). Informed consent was waived for this group because all data were retrospectively collected. In the second group, 48 consecutive patients who underwent unenhanced abdominal LDCT from May 2020 to October 2020 were included, and written informed consent was obtained from all patients prior to enrollment in our study because all data were prospectively collected. We did not observe any unexpected events, including technical failure and unstable breath holding among these 48 patients. Therefore, all patients were enrolled in this study (LDCT group). Detailed patient information was obtained from the respective medical records to document the demographics of our sample.

### Imaging technique

All unenhanced abdominal CT examinations were performed using a fast kilovoltage-switching dual-energy CT scanner (Revolution CT; GE Healthcare, Milwaukee, WI, USA) that was used in single-energy CT mode. The CT parameters were as follows: X-ray tube voltage, 120 kilovolt peak (kVp); noise index, 7.0 Hounsfield unit (HU) in SDCT group and 14.0 HU in LDCT group [12, 13, 17] based on 5-mm reconstruction thickness; rotation time, 0.5 s; pitch, 0.508:1; table speed, 81.3 mm/s; and detector configuration, 128 × 0.625 mm in both groups.

For the SDCT group, raw data were reconstructed with adaptive statistical iteration reconstruction-Veo (ASiR-V; GE Healthcare) of 40% with 5-mm section thickness and 0% overlap. In contrast, raw data for the LDCT group were reconstructed with DLIR (TrueFidelity™; GE Healthcare) at high strength level with 5-mm section thickness and 0% overlap. In this study, we used a high strength level of DLIR because this level has been proved to strongly reduce background noise compared with low and middle strength levels of DLIR and ASiR-V in several studies [10–13, 18]. The CTDI_vol_, size-specific dose estimates (SSDE), and dose-length product (DLP) were recorded from the dose report.

### Quantitative image analysis

A radiologist (T.M. with 2 years of post-training experience in interpreting abdominal CT images) measured the mean CT numbers of the liver, pancreas, spleen, kidney, abdominal aorta, inferior vena cava, and portal vein using a commercially available DICOM viewer by placing ROI on axial images. The CT numbers of the liver were measured using an ROI in the anterior segment, while carefully avoiding large vessels, bile ducts, focal lesions, and artifacts. The CT numbers of the pancreas were measured using an ROI, while carefully avoiding the main pancreatic duct, visible vessels, focal lesions, and artifacts. Furthermore, the CT numbers of the spleen and kidney were measured using an ROI, while carefully avoiding visible vessels, focal lesions, and artifacts. The CT numbers of the abdominal aorta at the level of the first lumbar vertebral body were measured using an ROI that incorporated as much of the vascular lumen as possible, devoid of vascular walls, calcification, thrombi, and artifacts. Finally, the CT numbers of the inferior vena cava at the level of the first lumbar vertebral body and the main portal vein were measured using an ROI that incorporated as much of the vascular lumen as possible, devoid of vascular walls, thrombi, and artifacts.

Background noise was defined as one standard deviation of the mean CT number at the homogeneous anterior abdominal subcutaneous fat tissue. The signal-to-noise ratio (SNR) of each anatomical structure was calculated by dividing the CT number of the corresponding anatomical structure by the background noise.

### Qualitative image analysis

Two radiologists (T.M. and T.K., with 2 and 4 years of post-training experience in interpreting abdominal CT images, respectively), who were unaware of the CT protocols, independently and randomly reviewed the images and graded the image quality with respect to the overall image noise and overall image quality using a 5-point rating scale as follows: 5, excellent; 4, good; 3, acceptable; 2, suboptimal; and 1, unacceptable.

The same two radiologists also independently and randomly graded the depiction of the liver, pancreas, gall bladder, spleen, stomach, kidney, adrenal gland, bladder, prostate (if patients were men), uterus (if patients were women), intestinal tract, urinary duct, abdominal aorta, portal vein, and inferior vena cava using a 5-point rating scale as follows: 5, excellent; 4, good; 3, acceptable; 2, suboptimal; and 1, unacceptable.

### Statistical analysis

Statistical analyses were performed using the SPSS statistical software package (version 24.0; IBM Corp., Armonk, NY, USA). The data were tested for normal distribution using the Shapiro–Wilk test. We used unpaired *t*-test or chi-square test to compare patients’ age, sex, height, body weight, and body mass index between the SDCT and LDCT groups. We used the Mann–Whitney *U* test to compare the CTDI_vol_, SSDE, DLP, CT numbers, background noise, SNRs, and confidence ratings for overall image noise, overall image quality, and depiction of abdominal structures between the SDCT and LDCT groups. *P* values of less than 0.05 were used to denote statistical significance.

Interobserver variability in qualitative analysis was assessed using the *ĸ* statistics. A *ĸ*-value of ≤ 0.20 was interpreted as slight agreement, 0.21–0.40 as fair agreement, 0.41–0.60 as moderate agreement, 0.61–0.80 as substantial agreement, and ≥ 0.81 as almost perfect agreement [19].

## Results

### Phantom study

**Fig. 2:**
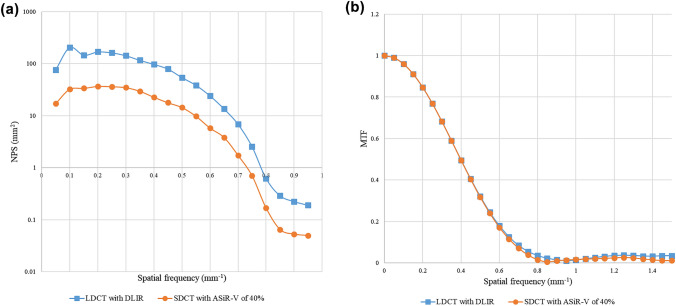
**a** Noise power spectrum (NPS) and **b** modulation transfer function (MTF) curves of low-dose CT (LDCT) reconstructed with high strength level of deep learning-based image reconstruction (DLIR) and standard dose CT (SDCT) reconstructed with adaptive statistical iteration reconstruction-Veo (ASiR-V) of 40%

NPS and MTF are shown in Fig. 2. The NPS curve analysis showed similar spatial frequency profile between LDCT protocol reconstructed with high strength level of DLIR and SDCT protocol reconstructed with ASiR-V of 40%, but the NPS value was higher in LDCT protocol reconstructed with high strength level of DLIR than in SDCT protocol reconstructed with ASiR-V of 40%. MTF_10%_ values were 0.65 in LDCT reconstructed with high strength level of DLIR and 0.65 in SDCT reconstructed with ASiR-V of 40%, that meant both of spatial resolution were almost same.

### Patients’ demographics and radiation dose

Patients’ demographics and radiation dose are summarized in Table 1. The SDCT group included 33 men and 25 women (mean age, 62.6 ± 16.0 years; age range, 18–90 years). Among them, three patients were status post hysterectomy, one was status post cholecystectomy, one was status post cystectomy, one was status post prostatectomy, and one was placed bilateral ureteral stent. The LDCT group included 26 men and 22 women (mean age 59.2 ± 16.0 years; age range 19–86 years). Among them, four patients were status post cholecystectomy, four were status post hysterectomy, and one was status post cystectomy. We did not assess the depiction of the corresponding structures in patients who had undergone surgery or treatment. No difference was noted in terms of patients’ age (*P* = 0.27), sex (*P* = 0.78), height (*P* = 0.97), body weight (*P* = 0.74), and body mass index (*P* = 0.72) between the SDCT and LDCT groups. The median CTDI_vol_ (14.8 mGy in SDCT group and 3.1 mGy in LDCT group; *P* < 0.001), median SSDE (20.0 mGy in SDCT group and 4.0 mGy in LDCT group; *P* < 0.001), and median DLP (810.9 mGy∙cm in SDCT group and 172.8 mGy∙cm in LDCT group: *P* < 0.001) were lower in the LDCT group than in the SDCT group. The average reduction rates of the CTDI_vol_, SSDE, and DLP were 78.9%, 80.5%, and 78.9%, respectively.

**Table 1:** Patients’ demographics and radiation dose

Parameter	SDCT group	LDCT group	*P* value
Age (y)	62.6 ± 16.0 (18–90)	59.2 ± 16.0 (19–86)	0.27
Men:Women	33:25	26:22	0.78
Height (cm)	162.0 ± 8.8 (145–184)	162.1 ± 11.4 (115–180)	0.97
Body Weight (kg)	57.8 ± 11.5 (36.7–85.9)	58.8 ± 11.7 (27.0–83.0)	0.74
Body Mass Index (kg/cm^2^)	22.0 ± 0.5 (15.8–29.7)	22.2 ± 0.5 (12.5–28.1)	0.72
CTDI_vol_ (mGy)	14.8 (11.3–17.9)	3.1 (2.8–3.6)	< 0.001
SSDE (mGy)	20.0 (17.1–23.0)	4.0 (3.7–4.4)	< 0.001
DLP (mGy∙cm)	810.9 (612.5–990.2)	172.8 (141.5–204.9)	< 0.001

Data are means ± 1 standard deviation with ranges in parentheses in patients’ demographics. Data are medians with interquartile ranges in parentheses in radiation dose

*SDCT standard-dose CT, LDCT low-dose CT, CTDI*_*vol*_ CT dose-index volume, *SSDE* size-specific dose estimates, *DLP* dose-length product

### Quantitative image analysis

The CT numbers, background noise, and SNRs are summarized in Table 2. Between the SDCT and LDCT groups, the CT numbers of almost all anatomical structures were statistically higher in the LDCT group compared to the SDCT group except for the liver (*P* < 0.001–0.048). However, the difference in the CT numbers ranged only between 0.9 and 3.4 HU. The background noise was observed to be lower in the LDCT group compared to the SDCT group (*P* = 0.02). Moreover, the SNRs of almost all anatomical structures were higher in the LDCT group than in the SDCT group except for the liver (*P* < 0.001–0.004). No difference was found in the CT number (*P* = 0.45) and SNR (*P* = 0.07) of the liver between the SDCT and LDCT groups.

**Table 2:** CT numbers and signal-to-noise ratios of the abdominal anatomical structures and background noise

Anatomical structure	SDCT group	LDCT group	*P* value
Liver			
CT number	58.6 (55.1–63.3)	60.3 (53.2–63.3)	0.45
SNR	9.9 (8.2–11.6)	11.3 (9.1–12.8)	0.07
Pancreas			
CT number	45.7 (42.6–48.8)	48.7 (43.5–51.7)	0.02
SNR	7.5 (6.8–8.9)	8.9 (7.8–10.3)	0.002
Spleen			
CT number	50.1 (48.3–52.3)	51.0 (49.4–54.4)	0.048
SNR	8.7 (7.8–9.8)	10.0 (8.3–11.3)	0.004
Kidney			
CT number	37.7 (35.8–39.5)	38.8 (37.4–40.8)	0.001
SNR	6.3 (5.8–7.3)	7.7 (6.3–8.7)	0.003
Abdominal aorta			
CT number	44.3 (40.4–46.5)	47.7 (44.9–50.4)	< 0.001
SNR	7.6 (6.2–8.6)	9.2 (7.7–10.7)	< 0.001
Inferior vena cava			
CT number	43.8 (40.1–47.3)	46.6 (43.5–49.7)	0.002
SNR	7.6 (6.1–8.7)	9.2 (7.5–10.3)	0.002
Portal vein			
CT number	41.3 (39.4–44.0)	43.6 (41.2–46.1)	0.002
SNR	7.2 (6.1–8.0)	8.6 (7.0–9.3)	0.003
Background noise	6.0 (5.1–6.4)	5.1 (4.6–6.1)	0.02

Data are medians with interquartile ranges in parentheses

*SDCT* standard-dose CT, *LDCT* low-dose CT, *SNR* signal-to-noise ratio

### Qualitative image analysis

The rating scores for overall image noise, overall image quality, and depiction of abdominal structure are shown in Table 3. The rating scores for the overall image noise were higher in the LDCT than in the SDCT group (*P* < 0.001 for both radiologists). No difference was observed in the rating scores for overall image quality between the SDCT and LDCT groups (*P* = 0.26 and 0.25 for radiologists 1 and 2, respectively). The *ĸ*-values ranged from 0.36 to 0.67, indicating fair to moderate agreement between the two radiologists.

**Table 3:** The rating scores for image noise, overall image quality, and depiction of abdominal anatomical structures in each reconstruction algorithm

Anatomical structure	SDCT group			LDCT group			*P* value	
	Radiologist 1	Radiologist 2	*κ* value	Radiologist 1	Radiologist 2	*κ* value	Radiologist 1	Radiologist 2
Overall image noise	4 (4–4)	4 (4–4)	0.67	4 (4–5)	5 (4–5)	0.39	< 0.001	< 0.001
Overall image quality	4 (4–4)	4 (4–4)	0.6	4 (4–4)	4 (4–4)	0.36	0.26	0.25
Depiction of anatomical structure								
Liver	5 (4–5)	5 (4–5)	0.68	5 (4–5)	5 (4–5)	0.45	0.13	0.11
Pancreas	4 (3–4)	4 (4–4)	0.69	4 (4–5)	4 (4–4)	0.58	0.28	0.06
Gall bladder	4 (3–5)	3 (4–4)	0.61	4 (3–5)	3 (4–4)	0.69	0.62	0.42
Spleen	5 (4–5)	5 (4–5)	0.71	5 (4–5)	5 (4–5)	0.43	0.43	0.30
Stomach	4 (4–5)	4 (4–4)	0.6	5 (4–5)	4 (4–5)	0.40	0.14	0.04
Kidney	4 (4–5)	4 (4–5)	0.65	5 (4–5)	4.5 (4–5)	0.38	0.04	0.046
Adrenal gland	4 (3–4)	4 (3–4)	0.66	4 (4–4)	4 (4–4)	0.56	0.09	< 0.001
Bladder	4 (4–5)	4 (3–4)	0.45	4 (4–5)	4 (4–4)	0.27	0.35	0.002
Prostate	4 (4–5)	4 (4–4)	0.39	4 (4–4)	4 (3–4)	0.78	0.03	0.47
Uterus	4 (4–5)	4 (3–4)	0.54	4 (3–5)	4 (4–5)	0.51	0.64	0.09
Intestinal tract	4 (3–5)	4 (3–4)	0.73	4 (4–4)	4 (4–4)	0.77	0.03	0.005
Urinary duct	4 (3–4)	4 (3–4)	0.84	4 (3–4)	4 (4–4)	0.69	0.03	0.004
Abdominal aorta	4 (4–5)	4 (4–5)	0.56	4.5 (4–5)	5 (4–5)	0.51	0.42	0.88
Portal vein	3 (2–4)	3 (3–4)	0.68	3 (3–4)	4 (3–4)	0.54	0.36	0.01
Inferior vena cava	5 (4–5)	4 (4–5)	0.63	4 (4–5)	4 (4–5)	0.50	0.69	0.58

Data are medians with interquartile ranges in parentheses

*SDCT* standard dose CT, *LDCT* low-dose CT

**Fig. 3:**
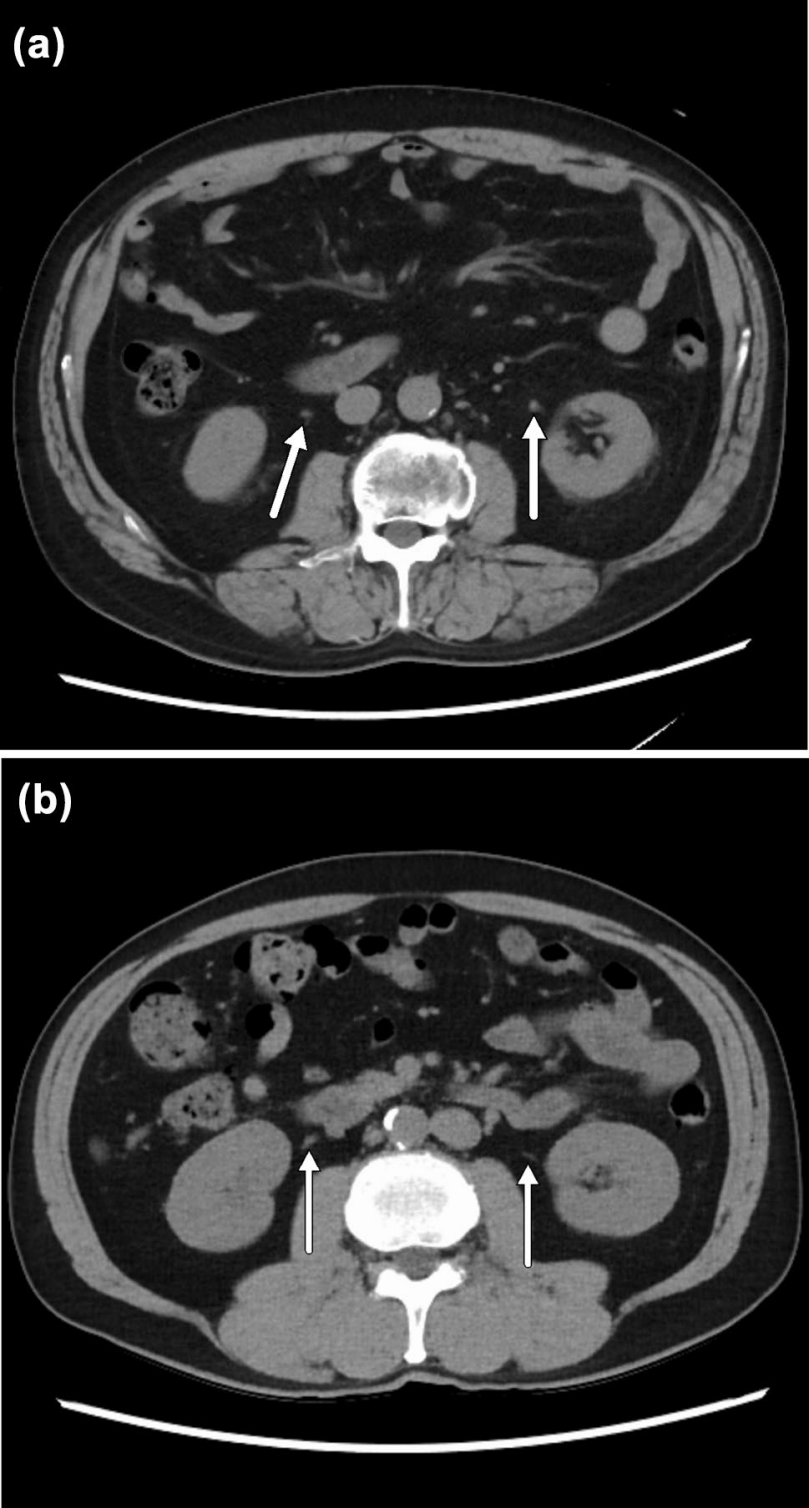
**a** Standard-dose axial image reconstructed with adaptive statistical iterative reconstruction-Veo of 40% in a 58-year-old man and **b** Low-dose axial image reconstructed with high strength level of deep learning-based image reconstruction in a 58-year-old man. CT image obtained using a low-dose protocol clearly shows urinary ducts (arrows)

**Fig. 4:**
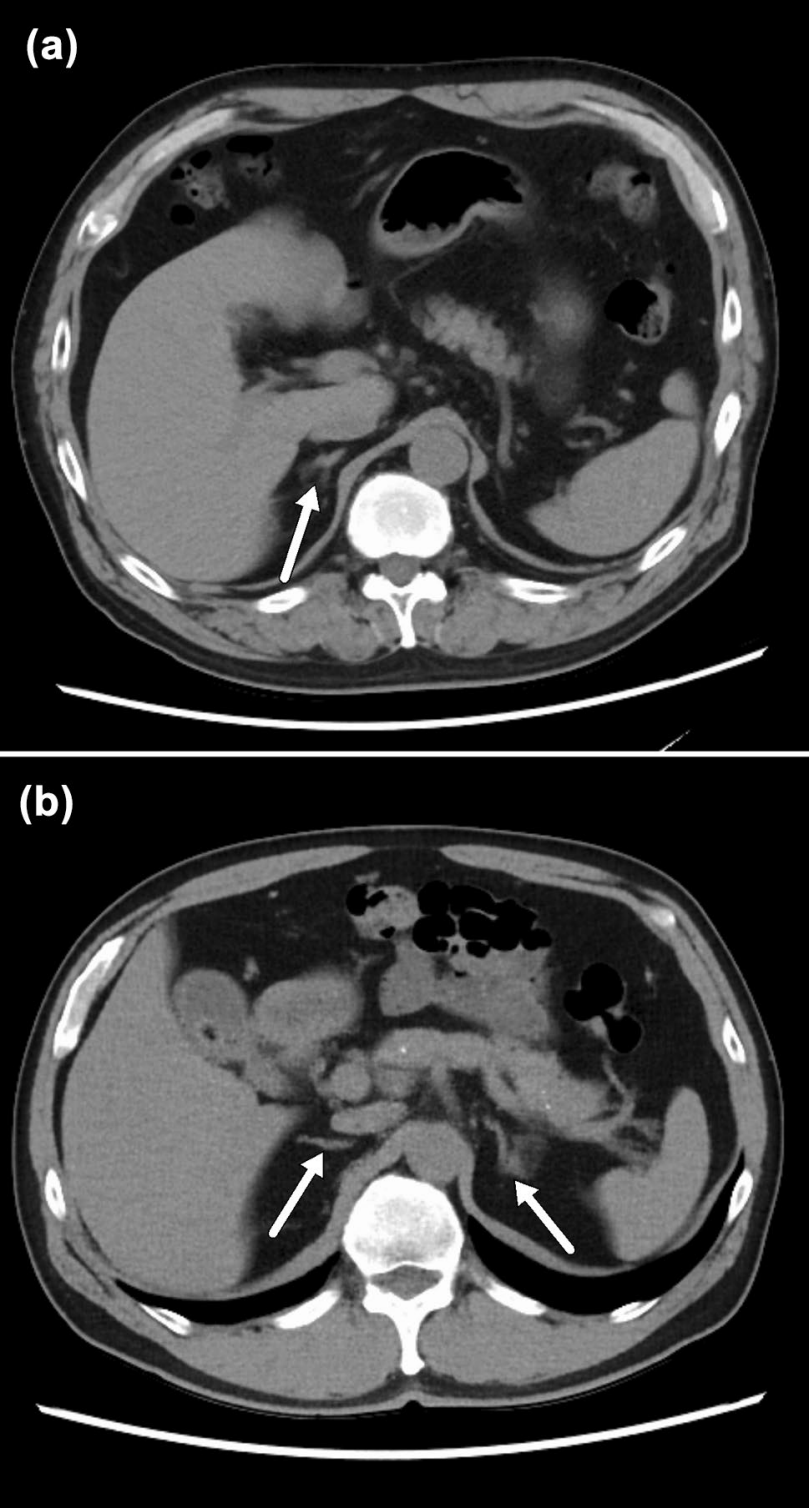
**a** Standard-dose axial image reconstructed with adaptive statistical iterative reconstruction-Veo of 40% in a 58-year-old man and **b** Low-dose axial image reconstructed with high strength level of deep learning-based image reconstruction in a 58-year-old man. CT image obtained using a low-dose protocol clearly shows the upper abdominal organs even for adrenal glands (arrows)

The depiction scores of the liver, pancreas, gall bladder, spleen, uterus, abdominal aorta, and inferior vena cava were not different between the SDCT and LDCT groups for both radiologists (*P* = 0.06–0.88). In contrast, the depiction scores of the kidney, intestinal tract, and urinary duct were higher in the LDCT group than in the SDCT group for both radiologists (*P* = 0.004–0.046) (Fig. 3), whereas the scores of the stomach, adrenal gland, bladder, and portal vein were higher in the LDCT group than in the SDCT group only for radiologist 2 (*P* < 0.001–0.04) (Fig. 4). The depiction score of the prostate was found to be higher in the SDCT group than in the LDCT group only for radiologist 1 (*P* = 0.03). The *ĸ*-values ranged from 0.27 to 0.84, indicating fair to almost perfect agreement between the two radiologists.

## Discussion

Our study has demonstrated that unenhanced abdominal LDCT protocol with DLIR algorithm allows > 75% radiation dose reduction in comparison to the SDCT protocol with hybrid-IR. In phantom study, the NPS value was higher in LDCT protocol reconstructed with DLIR algorithm than SDCT protocol reconstructed with ASiR-V of 40%. Additionally in human clinical study, LDCT protocol with DLIR algorithm was maintaining good image quality and depiction of abdominal anatomical structures.

To verify the utility of DLIR in LDCT protocol, several phantom studies had performed by comparing hybrid- or model based IR algorithms and showed that DLIR demonstrate less noise and higher detectability in same dose reduction level compared with the other reconstruction algorithms [20, 21]. In our phantom study, the NPS value was higher in LDCT reconstructed with DLIR than in SDCT reconstructed with ASiR-V of 40% though spatial resolutions were almost same. Nevertheless, overall image quality and noise level of LDCT protocol with DLIR algorithm was equivalent or better than those of SDCT protocol with ASiR-V of 40% in human clinical study. The following points could be given as reasons, the trivial discrepancy of NPS between the two protocols, preserved spatial resolution, and maintained image texture. As a result, radiologists’ subjective acceptance was kept even in LDCT with DLIR protocol.

DLIR managed to reduce image noise in the LDCT protocol in this study and this finding is consistent with the results of a previous study which compared image noise in the LDCT protocol with hybrid-IR and DLIR in patient [13, 14]. The vendor-specific DLIR was developed with high-quality FBP datasets to learn how to differentiate noise from signals and to suppress noise without changing the image texture [22]. In previous studies, SDCT images reconstructed with DLIR were superior to SDCT images reconstructed with hybrid-IR technique in terms of image noise, SNR, and contrast-to-noise ratio [10, 11, 18]. Both quantitative and qualitative image noise were significantly reduced in the LDCT group compared with the SDCT group in this study. In general, one of the most crucial issues in the LDCT protocol is noise reduction; however, it can be suggested that the development of DLIR has allowed us to overcome this issue. In addition, the overall image quality of the LDCT group was comparable with the SDCT group. We believe that DLIR can achieve effective noise reduction but also maintain high levels of overall image quality even in the LDCT protocol.

The depiction of abdominal structures in the LDCT group was equal to or greater than in the SDCT group. Maintaining a normal organ depiction is a minimum requirement for clinical use; thus, we believe that LDCT images reconstructed with DLIR can satisfy this requirement. The notable point is that even small or narrow organs, such as the adrenal gland and the urinary duct, were clearly depicted in the LDCT group (Figs. 3 and 4). Indeed, Noda et al*.* [13] have already reported that there were no differences in lesion detectability between the SDCT images reconstructed with hybrid-IR and LDCT images reconstructed with high strength level of DLIR in contrast-enhanced whole-body CT. Similarly, Singh et al*.* [17] reported that all clinically important lesions could be detected on contrast-enhanced LDCT with DLIR. Moreover, Jensen et al. [14] reported the utility of contrast-enhanced LDCT with DLIR for detecting liver lesions. Although we did not evaluate the diagnostic ability, we believe that our results provide solid evidence for the feasibility of unenhanced abdominal LDCT using DLIR. Additionally, our study has the superiority compared with the previous studies in that we can show the feasibility of unenhanced CT, because unenhanced CT is more broadly used in clinical setting than contrast-enhanced CT.

DLIR has only recently become available. However, the hybrid-IR technique is still the mainstream for CT image reconstruction. The application of the hybrid-IR technique to the LDCT protocol had been considered; therefore, multiple studies have already investigated the efficiency of this method [6–8, 23–25]. Most of the studies concluded that LDCT reconstructed with the hybrid-IR technique could maintain an efficient image quality but undermine spatial resolution and the detectability of low-contrast lesions compared to SDCT reconstructed with FBP. Thus, the clinical use of abdominal LDCT reconstructed with hybrid-IR had been limited. Our study suggests that the advent of DLIR can improve LDCT image quality and make it comparable, or even superior, to the image quality of SDCT reconstructed with hybrid-IR technique. The clinical application of LDCT was expected in the scene like screening.

This study has several limitations. First, the study population was relatively small, and our investigation was carried out at a single institution. Second, the patients’ body size was small. Third, we were not able to compare lesion detectability between the SDCT and LDCT groups because we could not define reference standard. Finally, we only used a CT scanner from a single vendor. Therefore, further clinical studies on larger populations are required to validate our results and evaluate lesion detectability of LDCT with DLIR protocol for other types of CT scanners.

## Conclusions

DLIR allows substantial radiation dose reduction of > 75%, and it significantly reduces the background noise. Additionally, this study reveals that DLIR can maintain adequate image quality and anatomical structure depiction in unenhanced abdominal LDCT.
